# Melt crystallization mechanism analyzed with dimensional reduction of high-dimensional data representing distribution function geometries

**DOI:** 10.1038/s41598-020-72455-z

**Published:** 2020-09-22

**Authors:** Hiroki Nada

**Affiliations:** National Institute of Advanced Industrial Science and Technology (AIST), 16-1 Onogawa, Tsukuba, 305-8569 Japan

**Keywords:** Materials science, Mathematics and computing

## Abstract

Melt crystallization is essential to many industrial processes, including semiconductor, ice, and food manufacturing. Nevertheless, our understanding of the melt crystallization mechanism remains poor. This is because the molecular-scale structures of melts are difficult to clarify experimentally. Computer simulations, such as molecular dynamics (MD), are often used to investigate melt structures. However, the time evolution of the structural order in a melt during crystallization must be analyzed properly. In this study, dimensional reduction (DR), which is an unsupervised machine learning technique, is used to evaluate the time evolution of structural order. The DR is performed for high-dimensional data representing an atom–atom pair distribution function and the distribution function of the angle formed by three nearest neighboring atoms at each period during crystallization, which are obtained by an MD simulation of a supercooled Lennard–Jones melt. The results indicate that crystallization occurs via the following activation processes: nucleation of a crystal with a distorted structure and reconstruction of the crystal to a more stable structure. The time evolution of the local structures during crystallization is also evaluated with this method. The present method can be applied to studies of the mechanism of crystallization from a disordered system for real materials, even for complicated multicomponent materials.

## Introduction

Controlling melt crystallization is fundamental in industrial processes, including semiconductor, ice, and food manufacturing^1,2^, and thus the crystallization mechanism is an important research subject. Melt crystallization is accompanied by an increase in the structural order over time. Therefore, understanding the time evolution of the structural order in a melt during crystallization is essential to elucidating the crystallization mechanism, although this is difficult to clarify experimentally.

Computer simulations, such as molecular dynamics (MD)^3^, are helpful tools for investigating the structure of condensed systems. MD simulations have been used to investigate crystallization mechanisms^4–9^ and the structure of a melt and its relationship to crystallization^10–12^. These MD simulation studies suggested that the short-range structure in a melt has a crystal-like order, which may be important in crystallization.

The structure of a crystal has long-range order, and thus to elucidate the crystallization mechanism, it is crucial to investigate the time evolution of the long-range order in the structure of a melt in addition to the short-range order^13^. The long- and short-range orders should be evaluated quantitatively to elucidate the mechanism in detail^14^. Moreover, it is desirable to use simple, generally used measurements to evaluate the long- and short-range orders to enable experimental verification of the crystallization mechanism.

Distribution functions, such as the pair distribution function (PDF), which shows the distribution of the distance between a pair of particles, *r*, have been used tentatively to represent the structural order in the structure of a melt. PDF provides a measure of the long- and short-range orders^15^. The short-range order can also be represented by the angular distribution function (ADF), which shows the distribution of the angle formed at a particle by its two nearest-neighbor particles, *θ*^15^. ADF provides features of the arrangement of nearest-neighbor particles, which corresponds to features of the short-range order. However, it is difficult to quantify the structural order represented by the PDF or ADF properly, and this cannot be done by visual inspection. Errington and Debenedetti quantified the PDF by integrating it for a certain interval of integration^13^, although it is preferable to quantify the PDF without introducing a parameter that can be defined arbitrarily.

Recently, a method for evaluating the structural similarity between different phases using a dimensional reduction (DR) technique, which was developed for machine learning and statistics, has been proposed^16^. In this method, high-dimensional data representing the geometry of the PDF or ADF for each phase is transformed into a two-dimensional (2D) data point by DR. The structural similarity between different phases is evaluated by the distance between 2D data points for those phases assuming that the structural similarity can be regarded as the geometrical similarity of the PDF or ADF. This method provided a reasonable description of the structural similarity for different crystal polymorphs and the amorphous phase of calcium carbonate^16^. Thus, we expect that the time evolution of the long- and short-range orders during crystallization can be evaluated quantitatively by applying this method to the geometry of the PDF or ADF at each moment during crystallization.

In this study, we demonstrate that the method based on the DR of high-dimensional data to a 2D data point has great potential for elucidating the mechanism of crystallization from a melt. The method was used to analyze the crystallization mechanism of a supercooled Lennard–Jones (LJ) melt, which was obtained with an MD simulation. The time evolution of the long- and short-range orders was evaluated by the DR of the geometries of PDF and ADF during crystallization. In addition, the time evolution of the distribution of local structures was evaluated by the DR of the geometry of the ADF created with each particle and its two nearest neighbors during crystallization. Based on these results, we discussed the applicability of our method for revealing the crystallization mechanism.

## Results and discussion

### Dimensional reduction

DR has been developed in machine learning and statistics^17–21^ for transforming original high-dimensional data into low-dimensional (usually 2D) data without significant loss of information^17,18^. When the 2D data obtained by DR of the original high-dimensional data are mapped onto a 2D plane, the 2D data points for similar original data are placed near to each other, and the 2D data points for different original data are placed far away from each other. Therefore, the similarities and dissimilarities among different high-dimensional data can be evaluated. So far, DR has been used to evaluate geometrical similarities or dissimilarities between different objects, such as surface recognition^22^, face recognition^23^, and surface matching^24^, in which DR was performed for high-dimensional data representing the geometry of each object. Recently, DR was used to evaluate the geometrical similarity of the PDF and ADF between different phases of calcium carbonate, and the results represented the structural similarity among those phases in real systems^16^.

**Figure 1:**
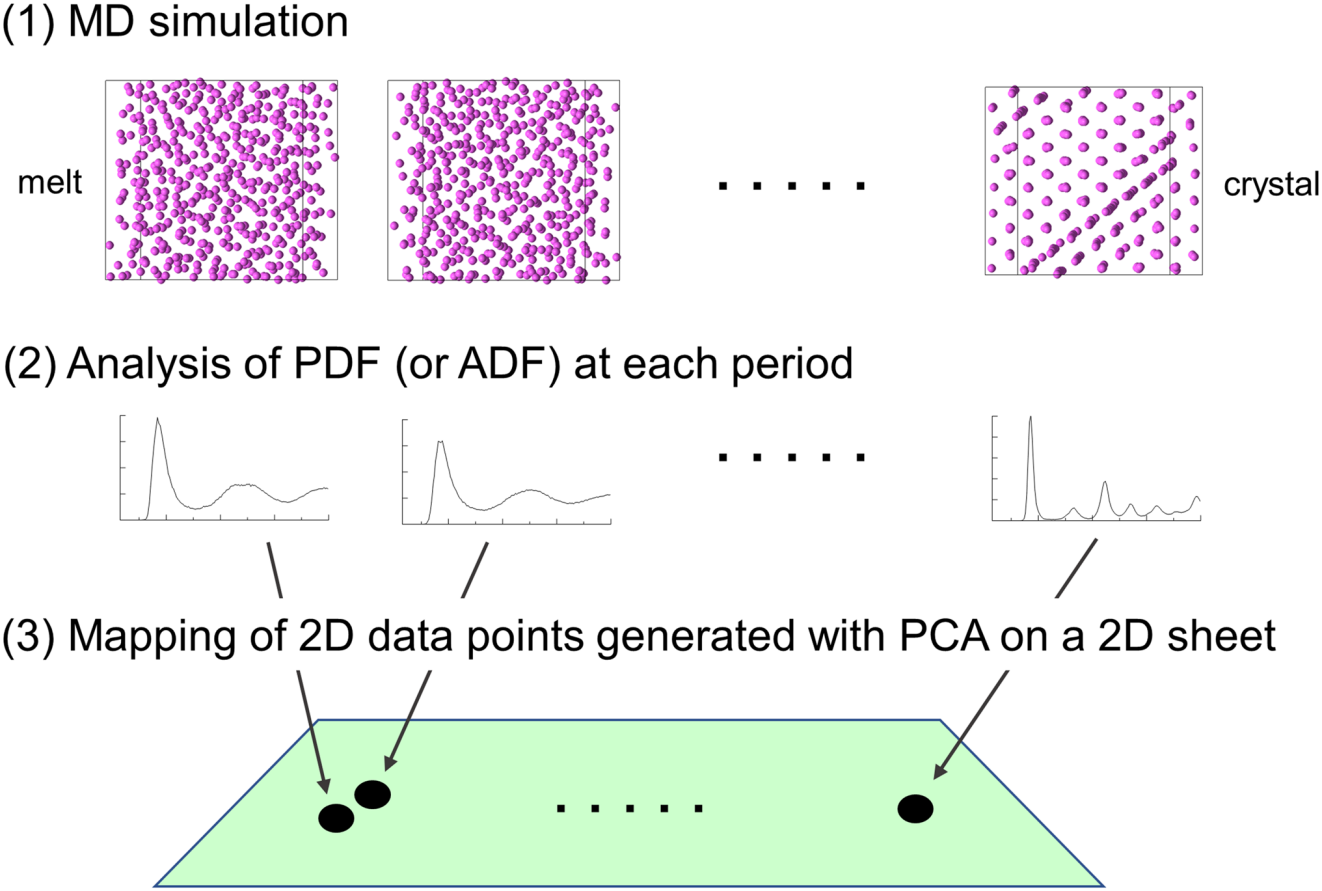
Outline of the procedure for evaluating the crystallization mechanism. (1) An MD simulation for crystallization of a supercooled LJ melt was performed. (2) Using the coordinates of LJ particles obtained during the MD simulation, the PDF and ADF were analyzed every 1 ps. Then, the time-averaged geometry of 20 consecutive PDFs (or ADFs) was created for every 20 ps. (3) Each time-averaged geometry of PDF (or ADF) was transformed into a 2D data point with PCA, and the 2D data point was mapped onto a 2D sheet formed by two principal eigen vectors (see text).

Figure 1 shows the outline of the procedure for evaluating the crystallization mechanism. The geometry of PDF (or ADF) was generated with an MD simulation of the crystallization of a supercooled LJ melt at each period in the simulation run and was transformed into a 2D data point using principal component analysis (PCA)^20^, which is a common DR algorithm. PCA requires a shorter computation time than other DR algorithms, such as multidimensional scaling^19^. All the 2D data points obtained with PCA during crystallization were mapped onto a 2D sheet. The structural similarity among different periods was evaluated by the distance between the 2D data points on the sheet; the shorter the distance was, the higher the similarity. In a previous study^16^, it was confirmed that the transformation of the PDF and ADF geometries into 2D data points with PCA provided qualitatively the same results as the transformation of them into three-dimensional data points with PCA. This result of the previous study suggests that the exactness of the features of the PDF and ADF geometries represented by 2D data points with PCA is sufficiently high. Mathematically, PCA performs an eigen decomposition for a covariance matrix produced by the original data points. The original data points are projected onto a 2D sheet formed by two principal eigen vectors, which correspond to the vectors in which the variance of the data points are the largest and second largest (PCA-1 and PCA-2, respectively)^20^. An explanation of the procedure for the transformation of a high-dimensional data into a lower-dimensional data point with PCA is shown in the Supplementary Information (Figure S1). PCA can extract important features of high-dimensional data having a linear data structure, whereas other DR methods, such as Sketch Map^25^, should be used if nonlinear data is examined.

Notably, a method of machine learning has also been used for earlier simulation studies on the local structure^26–28^, chemical reactions^29^, and cluster formation^30^ of condensed systems. However, to my knowledge, this study is the first to utilize DR for evaluating the crystallization mechanism.

### Crystallization mechanism

**Figure 2:**
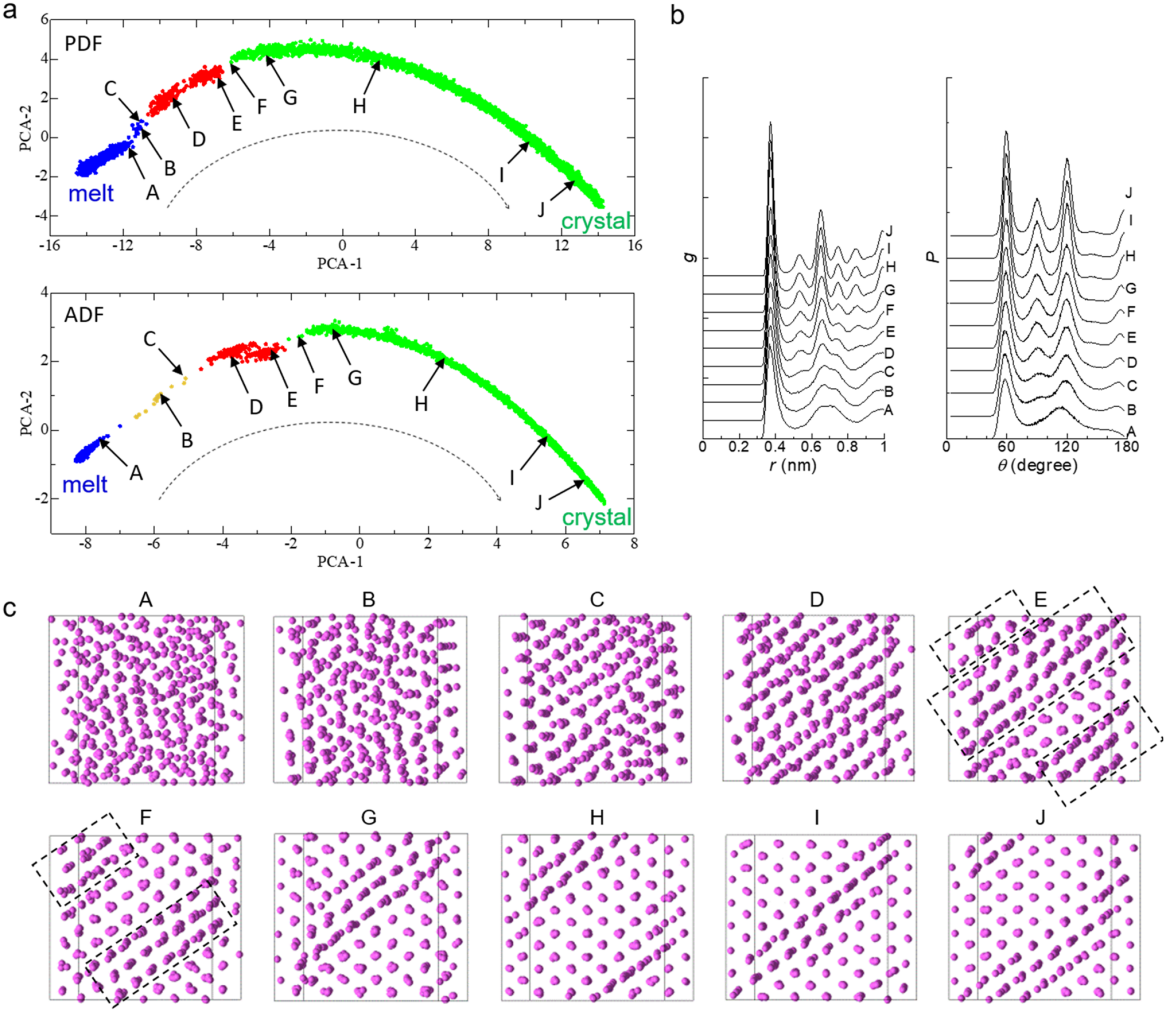
Results analyzed for the system with 500 particles. (**a**) 2D sheets onto which 2D data points of PDFs and ADFs, which were created with PCA, in different periods of the simulation are mapped. PCA-1 and PCA-2 are principal eigen vectors (see text). Colors indicate the clustering of the 2D data points using DBSCAN^31^ with epsilon, *ε*, of 0.4 and a minimum sample number, *N*_*min*_, of 15. The orange data points shown on the 2D sheet for the ADF are not included in any of the three groups. (**b**) PDF, *g*, as a function of *r* and ADF, *P*, as a function of *θ* for data points A‒J. The functions for points A‒J were obtained as the average of 20 functions for periods of (A) 25.488‒25.490 ns, (B) 25.510‒25.512 ns, (C) 25.516‒25.518 ns, (D) 25.768‒25.770 ns, (E) 25.976‒25.978 ns, (F) 25.998‒26.000 ns, (G) 26.198‒26.200 ns, (H) 27.198‒27.200 ns, (I) 29.198‒29.200 ns, and (J) 31.198‒31.200 ns. (**c**) Snapshots of the particles in the system at the end of the periods for points A‒J.

Figure 2a shows 2D sheets onto which 2D data points created by the DR of the PDF and ADF geometries during crystallization are mapped. The 2D data points at the beginning and end of the simulation are located at the left and right edges of the sheet, respectively. That is, the structural change from the initial melt to its crystalline form is represented as the arrangement of the 2D data points from the left- to right-hand side on the 2D sheets. The 2D data points were divided into at least three groups, indicated by the colors, as a result of clustering of the data points using the density-based spatial clustering of applications with noise (DBSCAN)^31^. This result suggests that three different structural states appeared during crystallization.

The rough structure of the system in each state was determined based on the PDF, ADF, and snapshots of particles in the system. Figure 2b shows the PDF and ADF for each data point A‒J shown in Fig. 2a, and Fig. 2c shows the corresponding snapshots. Point A corresponded to the melt, points D‒J to the crystal, and points B and C to the intermediate state between the melt and the crystal. Although points B and C on the 2D sheet for PDF were included in the first group by the clustering, they were included in a separate island of 2D data points adjacent to the main island of the 2D data points for the first group. These results show that the structural change represented by the abrupt shift of the 2D data points from the first to second groups corresponded to the nucleation of a crystal from the melt.

Because the supercooling was large, the subsequent growth of the crystal after nucleation occurred so quickly that the crystal grown could not ideally fit the system. Hence, the crystal had a partly distorted lattice structure (regions enclosed by dotted lines in the snapshot for point E). Therefore, the reconstruction subsequently occurred from the crystal with a distorted lattice structure to that with an energetically more stable structure in which the distortion was partly relaxed (regions enclosed by dotted lines in the snapshot for point F). The shift of the data points from the second and third groups corresponded to this reconstruction. The continuous shift of the data points in the third group toward the right edge of the sheet corresponded to the relaxation of the crystal structure after the reconstruction. The present method detected the crystal nucleation and the reconstruction, both of which are essential activation processes representing the crystallization mechanism.

The appearance of the 2D data points for the first group in a wide region along the PCA-1 axis suggests that the structure of melt varied continuously toward that of the crystal, and the crystal nucleation occurred when the melt structure approached as close as possible that of the crystal. The space between the 2D data point regions for the first and second groups was narrower for the PDF than for the ADF, indicating that the long-range order in the melt structure approached that in the crystal structure more closely than the short-range order did. Thus, the present method suggests that the increase in the long-range order associated with crystallization is quantitatively not the same as that for the short-range order. Notably, Russo and Tanaka suggested that time evolution of translational order was not the same as that of orientational order during crystallization^32,33^, which seems to be qualitatively consistent with the above suggestion by the present method. They also suggested that an increase in the orientational order was a trigger of crystallization rather than that in the translational order^33^.

**Figure 3:**
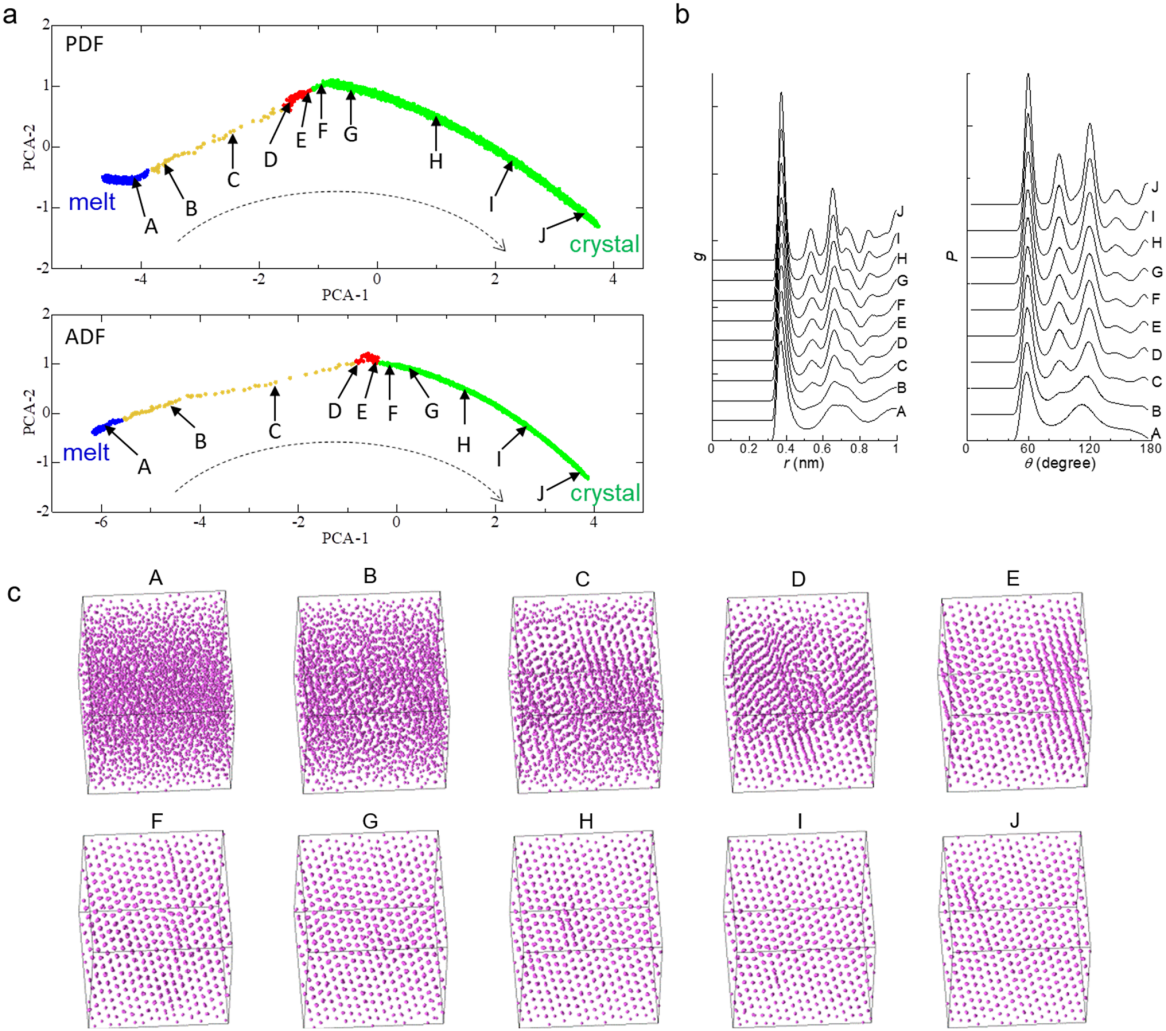
Results analyzed for the system with 4,000 particles. (**a**) 2D sheets onto which 2D data points of PDFs and ADFs, which were created with PCA, in different periods of the simulation are mapped. PCA-1 and PCA-2 are principal eigen vectors (see text). Colors indicate the clustering by DBSCAN^31^ with *ε* = 0.4 and *N*_*min*_ = 15. Orange data points in the 2D sheets are not included in any of the three groups. (**b**) PDF, *g*, as a function of *r* and ADF, *P*, as a function of *θ* for data points A‒J. The functions for points A‒J were obtained as the average of 20 functions for periods of (A) 18.209‒18.211 ns, (B) 18.243‒18.245 ns, (C) 18.245‒18.247 ns, (D) 18.247‒18.249 ns, (E) 18.255‒18.257 ns, (F) 18.258‒18.260 ns, (G) 18.277‒18.279 ns, (H) 18.442‒18.444 ns, (I) 18.570‒18.572 ns, and (J) 18.700‒18.702 ns. (**c**) Snapshots of particles in the system at the end of the period corresponding to points A‒J. The simulation for the large system was performed at 74 K.

To verify the crystallization mechanism suggested by the present method, the simulation and data analysis were also performed for a system consisting of 4,000 particles (the “large system”). The results are shown in Fig. 3. As in the small system consisting of 500 particles, the 2D data points were divided into groups for the melt, the crystal with a distorted structure, and the crystal with a more stable structure. The space between the 2D data point groups for the melt and the crystal with a distorted structure for the large system was also narrower for the PDF than for the ADF. Therefore, the results confirmed the validity of the crystallization mechanism obtained with the present method, which was qualitatively independent of the size of the system.

The change in the structure of the melt during crystallization was not identical in the two systems. Specifically, the 2D data point region for the first group was much narrower for the large system than for the small system, and the space between the 2D data point regions between the first and second groups was much wider for the large system than for the small system. These differences might originate from the difference in the structure of the melt, which could be affected by the system size. Strictly, the sizes of the present systems were not enough large to neglect the effect of the system size on the crystallization mechanism. However, the purpose of this study was to demonstrate the applicability of the present method to studies on the crystallization mechanism, but not to reproduce a crystallization process in real systems. It should be noted that even if the simulation results were affected by the size of the system and the periodic boundary conditions, the crystallization mechanism presented here is expected to apply to some crystallization phenomena in real systems, for example, poly-crystallization.

**Figure 4:**
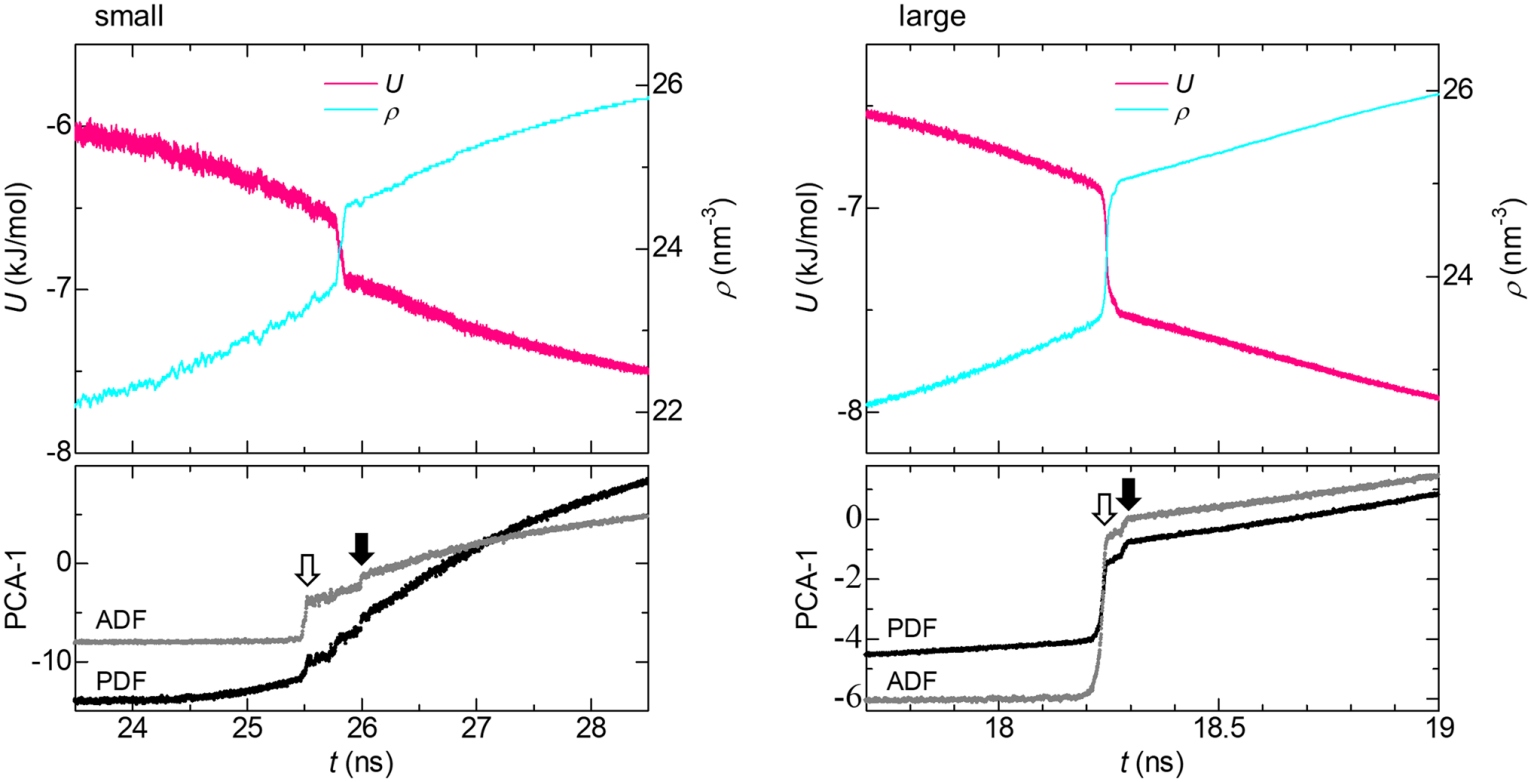
*U* and *ρ* as functions of *t* for the small (500 particles) and large (4,000 particles) systems. The lower panels show the values of PDFs and ADFs represented by one-dimensional (1D) data points as functions of *t*. The values were obtained by the transformation of the PDFs and ADFs into 1D data points along the principle eigen vector, PCA-1, with PCA.

Figure 4 shows the potential energy, *U*, and *ρ* as functions of time, *t*, for both the small and large systems. Abrupt changes in *U* and *ρ* associated with crystallization are seen around *t* = 25.8 ns for the small system and around *t* = 18.2 ns for the large system. However, the crystal nucleation and the reconstruction are barely visible in those functions. This difficulty in recognizing detailed structural evolution by visual inspection of those functions supports the great potential of the present method for evaluating complicated structural evolution processes that are hard to detect by conventional analytical methods.

In the lower panels of Fig. 4, one-dimensional (1D) data points into which were obtained by the transformation of PDFs and ADFs with PCA are also shown as functions of *t*. Abrupt changes, which corresponded to the nucleation (open arrows) and reconstruction (solid arrows), are seen even in those functions for the 1D data points. This result also supports the great potential of the present method for elucidating the crystallization mechanism.

### Time evolution of local structure distribution during crystallization

In principle, the ADF can be defined independently for each particle with its two nearest neighbors. The ADF defined for each particle with its two nearest neighbors (the “local ADF”) represents the local structural order. Therefore, the time evolution of the distribution of local structural orders during crystallization can be evaluated, which provides more detailed information on the crystallization mechanism, by creating a 2D sheet onto which the 2D data points for all local ADFs in each period are mapped.

**Figure 5:**
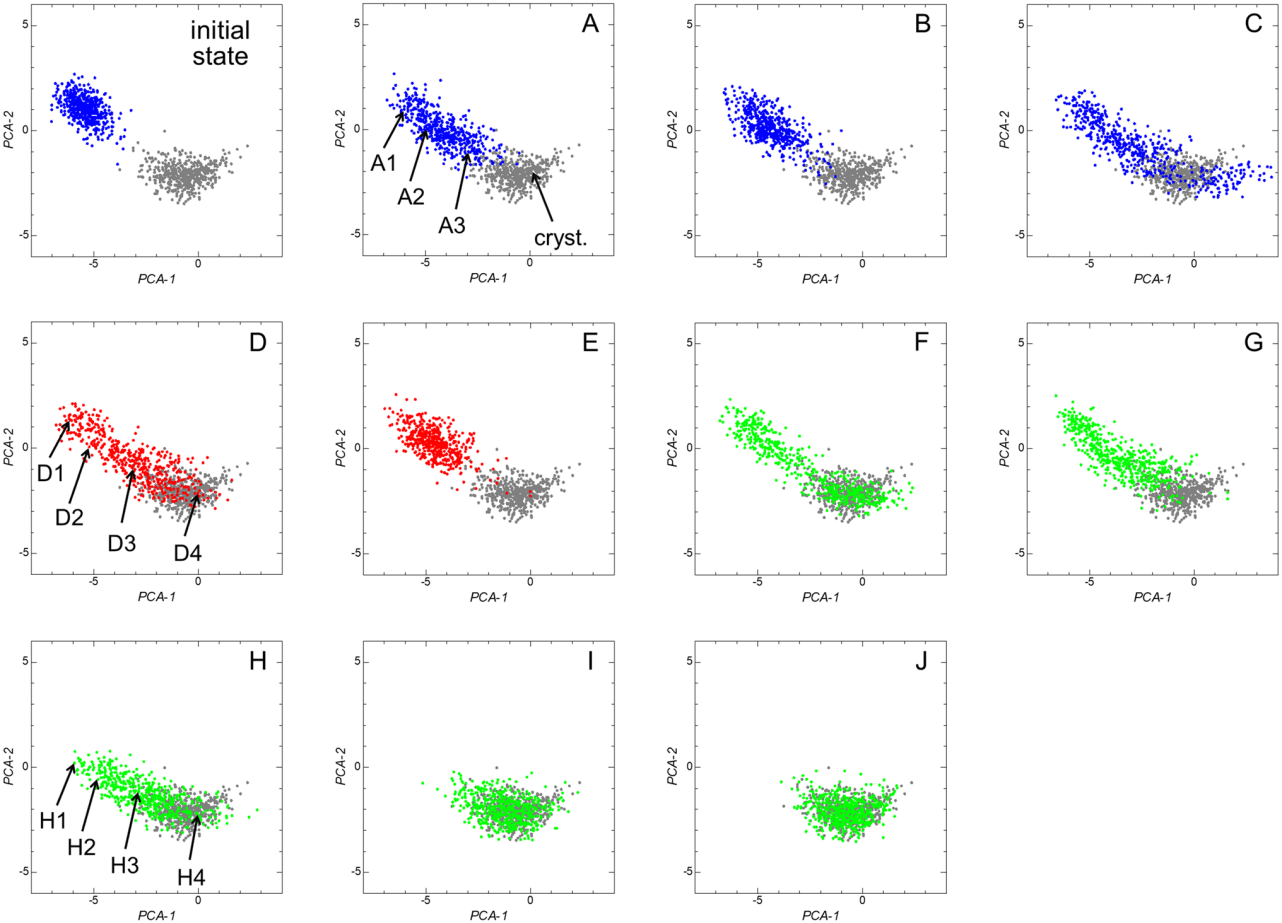
2D sheets onto which the distribution of the 2D data points for the local ADFs in the small system are mapped for the initial state and points A‒J. The 2D data points in the final state are also shown as gray dots on each 2D sheet.

Figure 5 shows 2D sheets onto which the 2D data points for the local ADFs in the small system for the initial state of the simulation (that is, the melt) and points A‒J are mapped. The 2D data points for the final state of the simulation (the crystal) are also shown as gray dots on all the 2D sheets. The 2D data points for points A and B are distributed not only in the melt region, but also in an intermediate region between the melt and crystal, suggesting that the melt for points A and B contained local structures with an intermediate structural order between the melt and crystal. For points C‒H, the 2D data points are distributed over the regions for the melt and crystal, and the intermediate region between them. These results strongly suggest that the increase in the local structural order associated with crystallization was spatially nonuniform.

**Figure 6:**
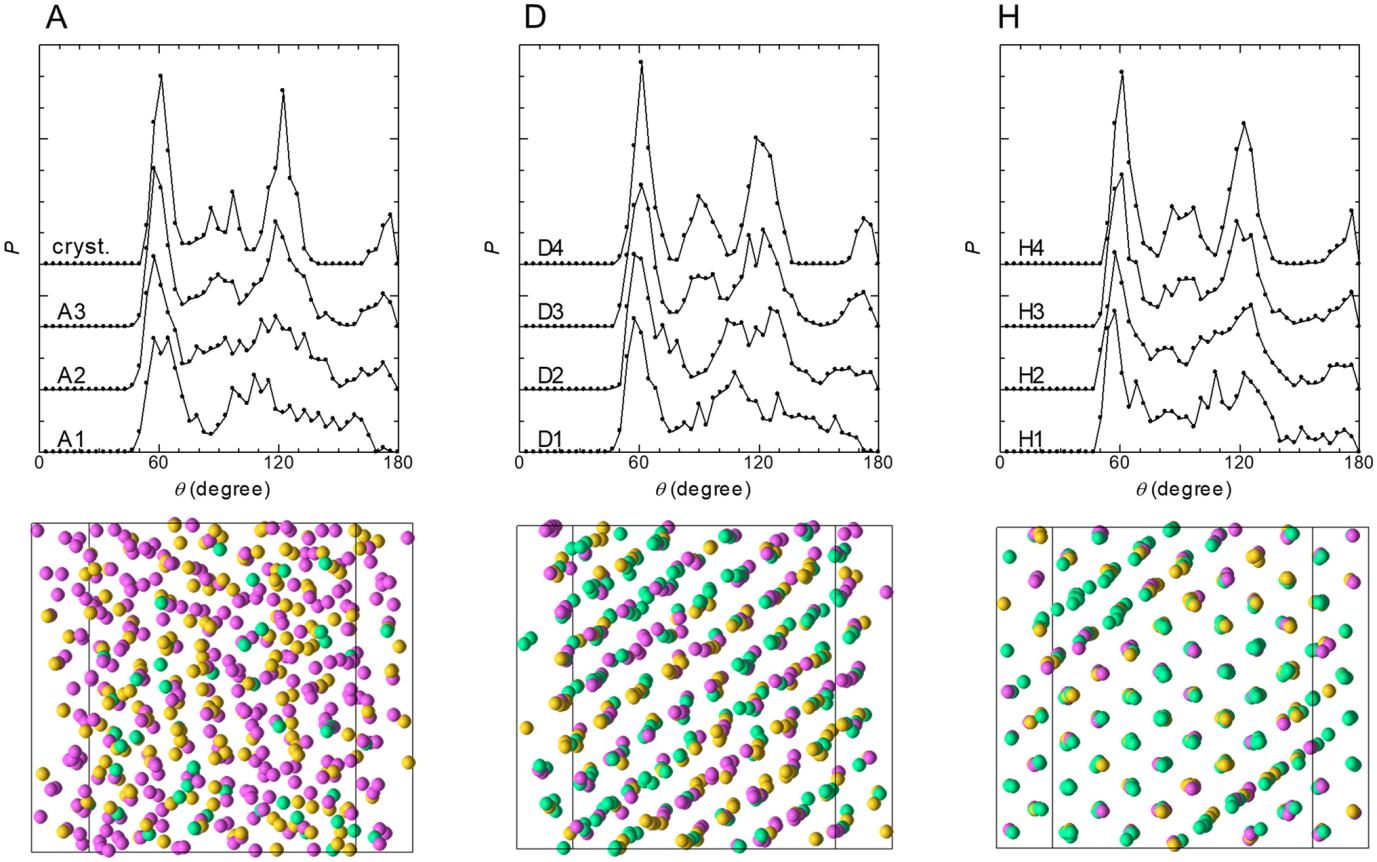
Geometries of the local ADFs for four selected 2D data points on each of the 2D sheets for points A, D, H in Fig. 5. A1, A2, D1, D2, H1, and H2 are in the melt region, A3, D3, H3 are in the intermediate region, and cryst., D4, and H4 are in the crystal region. The lower panels show snapshots of particles in which the particles creating the local ADFs for the melt, crystal, and intermediate regions are shown in magenta, yellow, and green, respectively.

The geometries of the local ADFs for four selected 2D data points on each of the 2D sheets for points A, D, and H in Fig. 5 (A1, A2, D1, D2, H1, and H2 are in the melt region, A3, D3, H3 are in the intermediate region, and cryst., D4, and H4 are in the crystal region) are shown in the upper panels of Fig. 6. The local ADFs in the crystal region had sharp peaks around *θ* = 60, 90, and 120°, whereas those in the melt region had a sharp peak around *θ* = 60° only. The local ADFs in the intermediate region had sharp peaks around both *θ* = 60 and 120°, whereas the peaks around *θ* = 90° were broad. The spatial distributions of the particles creating the local ADFs for the melt, crystal, and intermediate regions in the system are shown by the colors in the snapshots of the particles in the lower panels in Fig. 6. The local structures for those three regions can hardly be distinguished by visual inspection of the snapshots. Thus, the present method has great potential for evaluating the time evolution of the local structure distribution during crystallization.

## Conclusions

A method of visualizing high-dimensional data using DR, which was recently proposed for evaluating the structural similarity between different phases^16^, was used to investigate the mechanism of melt crystallization. The time sequence of the 2D data points created by the DR of the geometry of the PDF or ADF, which were obtained with an MD simulation of a supercooled LJ melt, was mapped onto a 2D sheet. The distribution of the 2D data points on the sheet suggested that crystallization occurred via the following activation processes: the nucleation of a crystal with a distorted structure and the reconstruction of the distorted crystal to a crystal with a more energetically stable structure. These activation processes were difficult to detect by visual inspection of the time evolution of the PDF, ADF, particle snapshots, *U*, and *ρ*.

The time evolution of the local structures in the melt during crystallization was also investigated by creating a 2D sheet onto which the 2D data points generated by DR of the local ADFs at each moment during crystallization were mapped to obtain more detailed information on the crystallization mechanism. The results showed that the increase in the local structural order associated with crystallization was spatially nonuniform, although it was impossible to distinguish the local structures with different structural order by visual inspection of the snapshots of the particles.

In summary, the application of the present method to simple, generally used measurements, such as the PDF and ADF, helped greatly to elucidate the mechanism of crystallization from a melt. We expect that the method will contribute to elucidating the crystallization mechanisms not only for a simple single-component system, but also for complicated multicomponent systems, such as for the crystallization of minerals^16,34–38^, clathrate hydrates^39–41^, and materials for devices^1,42–44^.

### Simulation methods

MD simulations were performed using a leapfrog algorithm with a time step of 10 fs for the small system and that of 2.5 fs for the large system^45^. The time step of 10 fs has been used in conventional MD simulations for LJ systems^46^. The fact that both simulations for the large and small systems provided qualitatively the same crystallization mechanism supports that the time step of 10 fs was sufficient for the purpose of this study. The values of parameters *ε* and *σ* in the LJ potential acting on each particle were set to those for Ar of 0.9961 kJ/mol and 0.3405 nm, respectively^47^. The temperature was maintained at 73 K and the pressure was maintained at 1 atm by a method proposed by Berendsen et al*.*^48^. The thermal and pressure bath constants were set to 0.1 and 2.0 ps, respectively. According to Golo and Shaitan^49^, the Berendsen thermostat may lead to a wrong kinetic energy distribution. However, it is known that the Berendsen thermostat provides collect dynamics of a system^50^. It was checked that the simulation with the Nosé-Hoover thermostat^51^ also showed the nucleation of a crystal with a distorted lattice structure and subsequent reconstruction of it to a more stable structure, which suggested qualitatively the same crystallization mechanism as described in this paper (Figure S2). The melting point, *T*_*m*_, of an Ar crystal at 1 atm in the LJ potential was 81.5 K^52^. The simulations were performed using DL_POLY 2.20^53^.

The simulation system was a cube consisting of 500 particles. Periodic boundary conditions were imposed in all three directions in the system. The structure of the melt was created as follows. All particles in the system were arranged into the lattice sites of a face-centered cubic crystal with the equilibrium density, *ρ*, at *T*_*m*_ and 1 atm. The system was heated at 500 K by a constant-volume 1 ns MD simulation to create a random arrangement of particles. The system was cooled to 100 K, which was higher by 23% than the melting point of 81.5 K, to create a melt structure with a constant-volume 10 ns MD simulation. The final configuration obtained by this MD simulation was used as the initial structure of the system for the crystallization simulation.

The simulation temperature of 73 K was lower than *T*_*m*_ by 10%. When the supercooling is large, crystallization may proceed via complicated multistage processes, starting with the formation of crystals with an incomplete structure in which distorted lattice and/or grain boundaries and/or point defects are created. The crystallization mechanism for such cases must be elucidated with a method that can evaluate the time evolution of the structural order in an impartial manner efficiently. Thus, we expect that the potential of the present method for elucidating the crystallization mechanism can be verified by examining crystallization during large supercooling.

The geometry of PDF was created using 500 data points at intervals of 0.002 nm from *r* of 0‒1 nm, and that of ADF using 200 data points at intervals of 0.9° from *θ* of 0°‒180°. For the calculation of *θ*, a selected pair of particles were judged to be the nearest-neighbor pair if the distance between the particles was less than 0.45 nm, which corresponded to *r* at which the first minimum of *g* for the melt appeared. DR of those functions and the creation of the 2D sheet were performed using Python 3.6^54^ and scikit-learn^55^, which is a set of Python modules for machine learning and data mining.

## Supplementary information

Supplementary file1

## Data Availability

The datasets generated during and/or analysed during the current study are available from the corresponding author on reasonable request.
