# Supplementary material for: Melt crystallization mechanism analyzed with dimensional reduction of high-dimensional data representing distribution function geometries
Source: Sci Rep. 2020 Sep 22;10:15465. doi: 10.1038/s41598-020-72455-z (PMC7508891; doi:10.1038/s41598-020-72455-z)
Supplement: Supplementary file 1 — Supplementary file1 [file 41598_2020_72455_MOESM1_ESM.pdf]

Supplementary Information

Melt crystallization mechanism analyzed with dimensional reduction of high-dimensional data representing distribution function geometries

Hiroki Nada*

National Institute of Advanced Industrial Science and Technology (AIST), 16-1 Onogawa, Tsukuba 305-8569, Japan.

*email: hiroki.nada@aist.go.jp

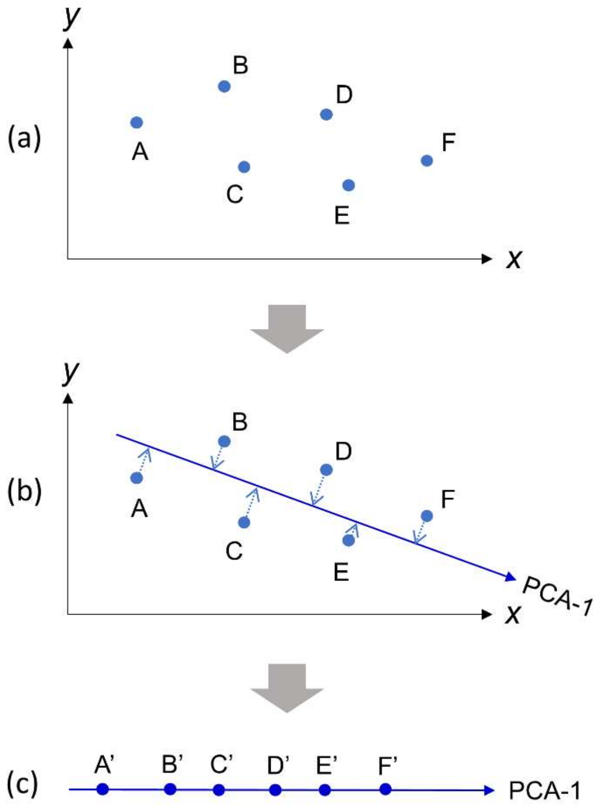

Figure S1. Explanation of the procedure for the transformation of high-dimensional data into lower dimensional data with PCA.[1] Here, as an example, the procedure for the transformation of two-dimensional (2D) data points into one-dimensional data points is schematically shown. (a) Distribution of original 2D data points (A, B, C, D, E, F) on the *x*-*y* plane. (b) Projection of the original data points onto an axis (PCA-1) along which the variance of the original data points is best captured. (c) Distribution of the projected data points (A', B', C', D', E', F') on the PCA-1 axis after DR. The data points that are placed near to each other on the *x*-*y* plane are placed near to each other even on the PCA-1 axis. The DR of higher-dimensional data than 2D data can also be done with the same procedure. Reproduced from H. Nada, *ACS Omega* **3**, 5789-5798 (2018). Copyright 2018 American Chemical Society.

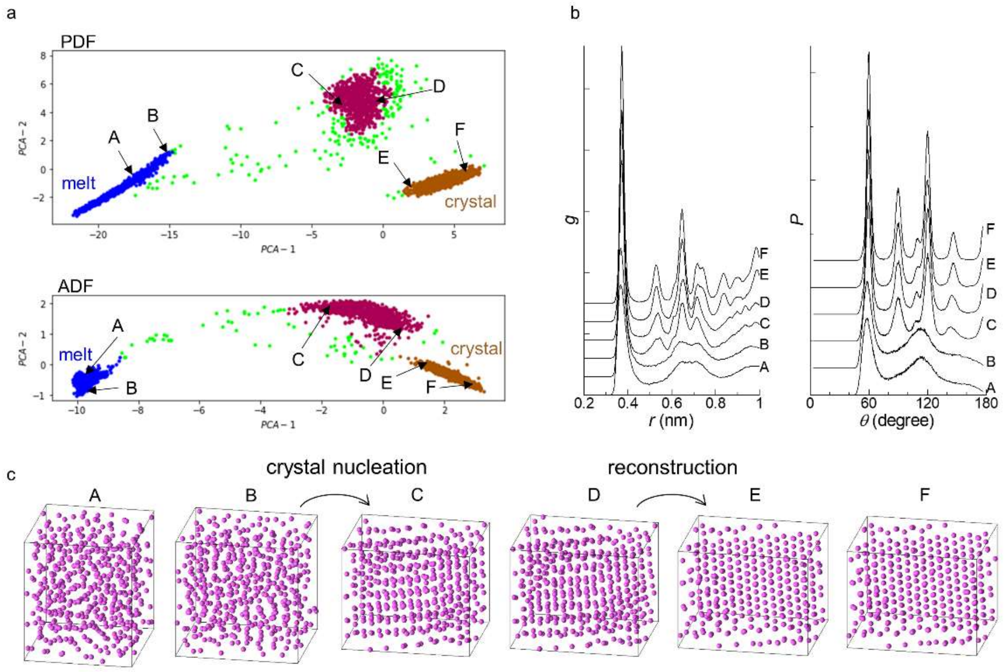

Figure S2. Results of a 10-ns MD simulation with the Nosé-Hoover thermostat[2] for the small system. The temperature was maintained at 73 K and the pressure was maintained at 1 atm. The thermal and pressure bath constants were set to 0.1 and 2.0 ps, respectively. The simulation was performed using the NσT ensemble in DL_POLY_2.20.[3] Computation was performed using a leapfrog algorithm.[4] The time step was 10 fs. (a) 2D sheets onto which 2D data points of PDFs and ADFs, which were created with PCA, in different periods of the simulation are mapped. Colors indicate the clustering of the 2D data points using DBSCAN[5] with epsilon, ε, of 0.4 and a minimum sample number, N_{min}, of 15. The greenyellow data points shown on the 2D sheets are not included in any of the three groups. (b) PDF, g, as a function of r and ADF, P, as a function of θ for data points A–F. The functions for points A–F were obtained as the average of 20 functions for periods of (A) 0.088–0.090 ns, (B) 1.334–1.336 ns, (C) 1.750–1.752 ns, (D) 3.030–3.032 ns, (E) 4.178–4.180 ns, and (F) 6.958–6.960 ns. (c) Snapshots of the particles in the system at the end of the periods for points A–F.

References

1. Nada, H. A new methodology for evaluating the structural similarity between different phases using a dimensionality reduction technique. *ACS Omega* **3**, 5789–5798 (2018).
2. Hoover, W. G. Canonical dynamics: Equilibrium phase space distributions. *Phys. Rev. A* **31**, 1695–1697 (1985).
3. Smith, W. & Forester, T. R. DL_POLY_2.0: a general-purpose parallel molecular dynamics simulation package. *J. Mol. Graph.* **14**, 136–141 (1996).
4. Allen, M. P. & Tildesley, D. J. *Computer Simulation of Liquids* (Clarendon Press, Oxford, 1989).
5. Ester, M., Kriegel, H.-P., Sander, J. & Xu. X. A density-based algorithm for discovering clusters in large spatial databases with noise. *Proceedings of the Second International Conference on Knowledge Discovery and Data Mining (KDD-96),* AAAI Press, 226–231 (1996).
